# Training needs of clinical research associates

**DOI:** 10.4103/2229-3485.71771

**Published:** 2010

**Authors:** Samyuktha Ajay, Arun Bhatt

**Affiliations:** *Clinical Operations, Feasibility and Site ID, Quintiles India, 5th Floor, Leela Business Park, M. V. Road, Andheri (E), Mumbai - 400 059, India*; 1 *Clininvent Research Pvt Ltd, A-103, Everest Chambers, Marol Naka, Andheri - Kurla Road, Andheri (E), Mumbai - 400 059, India*

**Keywords:** Clinical research associates, clinical research training, modules, performance, roles, topics

## Abstract

Clinical research is a relatively new field in our country that has seen very rapid growth in the last few years. Availability of personnel appropriately trained to the specific requirements of the role they will perform in clinical research is critical for capacity expansion. Our study attempts to understand the specific areas of knowledge and skills that are important for the role of a clinical research associate. The survey was conducted among clinical research professionals from industry and academia who had more than five years of clinical research experience and held important decision making positions in clinical research (stakeholders). The survey questionnaire was designed as a matrix of various clinical research roles on the y-axis and six knowledge modules and eight skills on the x-axis. Respondents were asked to rate the importance of the knowledge /skills to the role of clinical research associates on a three point scale. In discussing results, a significant response was considered to be 50% or greater positive response from the total group. The significant findings were that general, ethics and clinical trial execution modules were rated as critical for the role of clinical research associate. Regulatory module was rated as important for the role. The other significant responses were that three of the sub-topics in the methodology module - framing a research proposal/protocol and experimental design, designing case report forms and EDCs and conducting PK studies - were rated as important and one sub topic in the data management and statistics module was rated as not important. All the skills except leadership skills were rated as critical for the role. The findings of our survey were in general on the lines of expectations of performance of the role. The general, ethics and clinical trial execution modules are critical knowledge areas for the role of a clinical research associate. No clear trends emerged for some of the other modules. Leadership skills were not rated as critical to the role. This kind of a survey gives a good direction when training curriculum has to be designed for specific roles in clinical research. However, there is a need to expand the sample size to fine-tune the knowledge and skills areas.

## INTRODUCTION

GATT compliance in 2005 and the positive regulatory changes that ensued have made India an increasingly attractive location for clinical research. We have witnessed a rapid growth in this sector in the last few years[1] with projections for increased requirements for personnel in the various roles like clinical research associate (CRA), investigators, site coordinators, data management personnel, statisticians etc.[2] These roles are very well differentiated in terms of their performance expectations and availability of training tailored to the specific role requirements will be critical to capacity expansion.[3]

The CRA has the very critical role of ‘monitoring’ a clinical trial, which includes that the rights and well-being of human subjects are protected, the reported trial data are accurate, complete, and verifiable from source documents and that the conduct of the trial is in compliance with the currently approved protocol/amendment(s), with GCP, and with the applicable regulatory requirement(s). About the training of monitors, the GCP guideline mentions only that monitors should be appropriately trained, and should have the scientific and/or clinical knowledge needed to monitor the trial adequately and that a monitor’s qualifications should be documented.

Since clinical research itself is a relatively new field in India, we considered it is important to understand the requirements of knowledge and skills for the diverse roles. This paper describes the survey findings of the knowledge and skills needed for the role of a clinical research associate. We conducted a survey among key stakeholders in clinical research professionals in the industry, who by virtue of their positions had directly or indirectly employed or worked with CRAs.

## MATERIALS AND METHODS

We chose to have the opinion of important stakeholders in clinical research on what they think are important knowledge and skill areas for a person performing the role of a clinical research associate. We looked for people who had more than five years of clinical research experience and held important decision making positions in clinical research (stakeholders). Forty eight such people were identified and they formed the population for this survey. The survey questionnaire was designed as a matrix of various clinical research roles on the y-axis and the areas of knowledge and skills on the x-axis.

Knowledge areas were further classified into six broad categories with sub-topics in each module as follows-

- General
- Ethics
- Regulations
- Methodology
- Data management and statistics
- Clinical trial execution

Skills were classified as-

- Leadership
- Teamwork
- Negotiation
- Conflict management
- Interpersonal skills
- Computing
- Presentation skills
- Communication skills

Each respondent graded the importance of the area of knowledge/skill for the role by grading it as:

3 - *Critical*- Performance of the role is not possible without knowledge of this area

2 - *Important*- Knowledge is important, but not critical to performance

1 - *Not important*- Knowledge is not important for performance in the current role

In discussing results, descriptive statistics will be used. A significant response was considered to be 50% or greater positive response from the total group as described by Stonier and Gabby in a similar study conducted by them.[4]

## RESULTS

Of the 48 questionnaires sent, we obtained 31 responses. Fifteen were from Indian CROs, six from multinational pharma companies, five from multinational CROs, three from Indian pharma companies and two from academic medical institutions [Table 1].

**Table 1:** Role profile of respondents

Head, clinical research	6
Head, Indian operations	6
Study managers	6
Senior managers	5
Head, business development	4
Medical directors	2
Head, department of pharmacology	1
Assistant Prof. pharmacology	1

The work experience distribution was as follows:

### Knowledge modules

**Figure 1:**
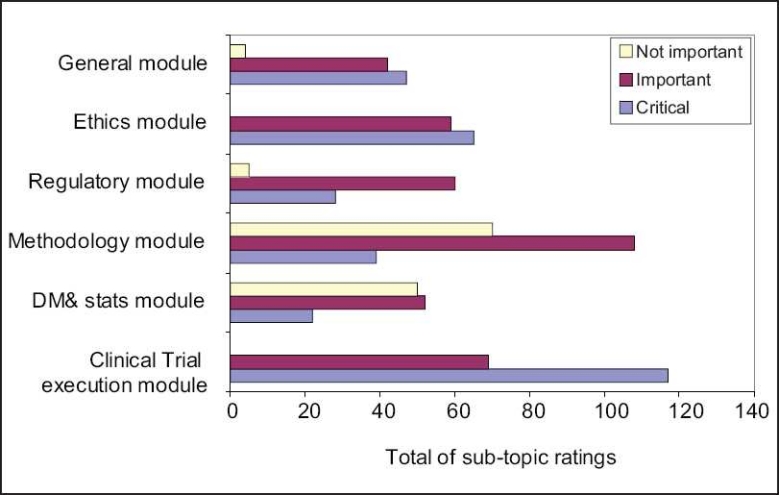
Responses for the role of clinical research associate (n=31)

A frequency distribution of grades for knowledge areas and skills are given in Tables 2 and 3 [Figure 1].

**Table 2:** Clinical research knowledge areas for clinical research associates

Areas of knowledge (n=31)		Critical	Important	Not important
General module	Scope of clinical research	11	16	4
	Orientation to pharmaceutical industry	18	13	0
	Drug development process	18	13	0
	Gradewise total of three topics in general module	47	42	4
Ethics module	Biomedical ethics- history and principles	15	16	0
	ICH GCP and national GCP guidelines	17	14	0
	EC composition and function- ICMR and ICH guidelines	16	15	0
	Informed consent process- principles and practice	17	14	0
	Gradewise total of four topics in ethics module	65	59	0
Regulations module	Regulations affecting CT for new product/generic registration in India including Schedule Y	9	21	1
	Regulations relating to IP labelling and import	10	19	2
	Regulations regarding safety and pharmacovigilance	9	20	2
	Gradewise total of three topics in regulatory module	28	60	5
Methodology module	Framing a research proposal/protocol and experimental design	4	19	8
	Writing investigators brochure	4	15	12
	Designing case report forms and EDCs	6	16	9
	Writing informed consent and patient information sheet	10	14	7
	Writing study reports and publication	6	14	11
	SOP writing	7	13	11
	Conducting PK studies	2	17	12
	Gradewise total of seven topics in methodology module	39	108	70
DM and stats module	Types of data and statistical tests for clinical trials	6	14	11
	Statistical considerations at the design, execution and analysis	5	15	11
	Data coding and cleaning	2	11	18
	Software considerations in data management	9	12	10
	Gradewise total of four topics in demand status module	22	52	50
Clinical trial execution	Monitoring a clinical study	23	8	0
	Project management in clinical research	18	13	0
	Legal issues in clinical research (legal, contractual, insurance, indemnity etc)	16	15	0
	Audits and inspection	18	13	0
	Clinical trial supplies management	22	9	0
	Pharmacovigilance and safety management	20	11	0
	Gradewise total of six topics in clinical trial execution module	117	69	0

**Table 3:** Clinical research skill areas for clinical research associates

Skills (n=26)	Rating		
	Critical	Important	Not important
Leadership skills	8	9	9
Team work	23	3	0
Negotiation skills	22	4	0
Conflict management	23	3	0
Interpersonal skills	22	4	0
Computing skills	18	8	0
Presentation skills	22	4	0
Communication skills	22	4	0

The key findings for knowledge and skills were as follows:

### Knowledge areas

There were 31 responses for knowledge areas and 26 responses were considered as evaluable for skills areas.

The general, ethics and execution modules were rated as ‘critical’ by 50% and more respondents. All the sub-topics in the clinical trial execution module were rated as critical.

The regulatory module and all the sub topics within were rated as “important” by 50% or more respondents.

The other significant responses were –

- Sub topics - Framing a research proposal/protocol and experimental design, designing case report forms and EDCs and conducting PK studies were rated as ‘important’
- Data coding and cleaning were rated as “not important’ by 50% or more respondents.

Ratings for the other modules and sub topics did not reach significance level.

### Skills

Data of 26 responders were considered as evaluable for the skills. All the skills except leadership skills were rated as ‘critical’ by more than 50% of respondents. Team work and conflict management received the highest number of “critical’ responses closely followed by negotiation, interpersonal, presentation and communication skills.

## DISCUSSION

Our survey suggests that for the CRA role, knowledge of the subtopics within the general, ethics and clinical trial execution modules is critical. These modules included most of the sub-topics that directly impact his critical role of ‘monitoring’ a clinical trial- including the ethical aspects of the rights and well-being of human subjects and the execution aspects that the reported trial data are accurate, complete, and verifiable.

There was no clear trend otherwise, with the regulatory module and all the sub topics within it being rated as ‘important’. This is rather surprising to find as compliance with GCP, and with the applicable regulatory requirement(s) is also an important aspect of the monitoring role. It was also interesting to note that while significant number of stakeholders rated the execution aspects of pharmacovigilance and safety management as ‘critical’, the regulations pertaining to the same were rated as ‘important’.

The only other survey done in India to understand training needs was done in 2003, to give direction to the curriculum development for courses proposed by the Academy for Clinical Excellence (ACE).[5] We were involved in this survey as executive curriculum committee members and active contributors for the set-up of curriculum for ACE. The population surveyed included investigators, CRO and pharmaceutical industry professionals and academia. The questionnaire captured the response as a rating of the importance of training in four modules of clinical research: clinical research regulations and environment, clinical research administration, clinical research methodology and applied clinical research. The methodology module received the highest rating, with 38% respondents rating it as important. However, this survey did not cover specific knowledge subtopics or skills for different roles.

The CRA is the central point of communication between sites and the sponsor and it was as expected to find communication skills, presentation skills, computing, interpersonal, conflict management, negotiation and team work rated as critical. It was surprising to find no significant trends in leadership skills for this role, which was probably because of the understanding and perception of the term leadership.

Although our survey had a small sample size, the trends that emerged match the performance expectations of the role, validating the study data. The three point grading we used to grade the importance of the areas of knowledge and skills requirements was probably not discriminatory enough. Hence there was equal weight given to critical and important grades for some subtopics for the CRA role. It was probable that the grading of ‘critical’ and ‘important’ were directionally similar and not characterized enough to avoid interchangeable use. Besides, we had a larger number of industry respondents and under-representation of academia, probably skewing the data to industry expectations. Our respondents belonged to organizations of various sizes and types and their own experiences would have colored their perception of training requirements of this role. Also, there were few areas that were grouped as knowledge areas, which could probably be better classified as competencies, eg, writing investigators’ brochure or designing case report forms and EDCs. Another limitation of our study was that we had not added behavioral indicators to describe each of the skills terms.

Survey was launched in 2006 and since then the clinical research industry has grown exponentially, with much technological advancement –e.g. most studies are now using e-CRFs. The role of the CRA has also evolved from the traditional role of compliance monitor to a new one of site relationship builder is being discussed with different competencies.[6] Zimmerman includes skills in using and troubleshooting hardware- computers, peripherals and other office apparatus and knowledge of multiple software programs- standard word processing database, project management, productivity programs and custom designed intranet software program as important competencies in preparing CRAs for the 21st century.[7]

The challenge to define the training requirements of this role can be understood from the predicament that even the association of clinical research professionals (ACRP), the leading certifier of CRAs for the last several years, is still trying to define what the minimum education and training requirements for CRAs ought to be.[8]

This kind of a survey gives a good direction when training curriculum has to be designed for specific roles in clinical research. However, there is a need to expand the sample size to fine-tune the knowledge and skills areas.

Disclaimer: All opinions expressed herewith are those of the authors and do not reflect the views of their organizations.
